# On the Evaluation of Diverse Vision Systems towards Detecting Human Pose in Collaborative Robot Applications

**DOI:** 10.3390/s24020578

**Published:** 2024-01-17

**Authors:** Aswin K. Ramasubramanian, Marios Kazasidis, Barry Fay, Nikolaos Papakostas

**Affiliations:** Laboratory for Advanced Manufacturing Simulation and Robotics, School of Mechanical and Materials Engineering, University College Dublin, Belfield, D04 V1W8 Dublin, Ireland; aswin.ramasubramanian@ucdconnect.ie (A.K.R.); marios_kazasidis@hotmail.com (M.K.); barryfay1999@outlook.com (B.F.)

**Keywords:** vision sensors, markerless tracking, collaborative robotics, data-fusion, human-tracking

## Abstract

Tracking human operators working in the vicinity of collaborative robots can improve the design of safety architecture, ergonomics, and the execution of assembly tasks in a human–robot collaboration scenario. Three commercial spatial computation kits were used along with their Software Development Kits that provide various real-time functionalities to track human poses. The paper explored the possibility of combining the capabilities of different hardware systems and software frameworks that may lead to better performance and accuracy in detecting the human pose in collaborative robotic applications. This study assessed their performance in two different human poses at six depth levels, comparing the raw data and noise-reducing filtered data. In addition, a laser measurement device was employed as a ground truth indicator, together with the average Root Mean Square Error as an error metric. The obtained results were analysed and compared in terms of positional accuracy and repeatability, indicating the dependence of the sensors’ performance on the tracking distance. A Kalman-based filter was applied to fuse the human skeleton data and then to reconstruct the operator’s poses considering their performance in different distance zones. The results indicated that at a distance less than 3 m, Microsoft Azure Kinect demonstrated better tracking performance, followed by Intel RealSense D455 and Stereolabs ZED2, while at ranges higher than 3 m, ZED2 had superior tracking performance.

## 1. Introduction

Industry 4.0 principles have been evolving in parallel with working environments that encapsulate human skills (e.g., cognition, decision making) and capabilities of robotic systems (e.g., dexterity, robustness, accuracy). Industry 4.0 technologies are on the advert of becoming an integral part of the current manufacturing ecosystem [1]. This causes multiple concerns about safety, ergonomics, and task optimisation [2], rendering the modelling of humans and their activities a critical aspect to be considered. Towards this direction, the utilisation of multiple sensors skeleton and Internet of Things (IoT) technologies, has gained popularity within manufacturing environments for various applications. The collection, transfer, and exchange of data from IoT devices via communication networks enable real-time interaction and cooperation among physical objects [3]. A series of virtual simulation-based solutions have been proposed, such as Digital Twin (DT), Cyber-Physical Systems (CPSs), and Digital Human Modelling (DHM), paving the way for fully digitising industrial shop floors [4,5,6].

The safety of operators within collaborative workspaces where they may share tasks with robots [7] is of paramount importance [8]. The design of these human–robot applications can be rendered more efficient with the utilisation of vision systems [9] as their technology enables the constant tracking and monitoring of human operators’ joints and the adaptive human–robot cooperation and interaction [10]. Typically, vision-based tracking solutions are widely categorised into marker-based and markerless. While marker-based tracking attains high accuracy, its increased cost, rigorous preparation requirements, and complexity have restricted its applicability [11]. On the other hand, markerless depth sensors with skeleton tracking capabilities have become increasingly popular due to their portability, generic applicability, and affordable cost [12]. These sensors exhibit Human Activity Recognition capabilities, by tracking the human pose and changes occurring in the environment and are applied to diverse research applications. However, Human Activity Recognition still remains a challenging area of research in computer vision [13]. 

Nevertheless, before considering vision systems as a viable option to operate in conjunction with or in lieu of ISO-certified sensor devices, such as safety camera systems, laser scanners, and proximity sensors [14], rigorous testing scenarios and methods are required to investigate their efficiency. The main goal would be to allow vision-based human tracking technologies to complement and work synergistically with built-in safety sensors that commercially available collaborative robots carry in order to overcome unforeseen circumstances to carry out complex tasks for instance shown in Ref. [15]. The identification or monitoring of specific safety features is required in industrial applications as per ISO/TS 15066 [16]. The specific standard provides a series of safety guidelines depending on the level of interaction and can be used complementarily to other ISO guidelines associated with robotic processes, such as ISO 10218-1:2011 [17], ISO 10218-2:2011 [18], and ISO 13855 [19,20]. Yet, it should be noted that the maturity and readiness of vision systems in industrial environments are still under review, since in some specific scenarios the detection of an operator may be prevented due to occlusions [21,22]. In these cases, using various types of sensors in conjunction with sensor fusion algorithms has been reported as a method to improve the overall perception of the process of human pose estimation for collaborative robotic applications [23].

In the present study, the main focus is placed on applications where understanding the complete body pose is crucial for effective human-robot cooperation. Three widely used vision sensors with high-depth accuracy were applied to detect human skeleton joints in two different poses, i.e., Azure Kinect (AK), Stereolabs ZED2, and Inter RealSense D455. Instead of detecting the pose of a perfectly planar object [24], the benchmarking in the present study involved the tracking of a human skeleton, aiming to investigate the performance of the sensors in a collaborative workspace in terms of accuracy and repeatability. The study estimates the coordinates of an operator’s joints at various depth levels from the cameras and compares them with the ground truth, calculating the average RMSE of the depth data. Furthermore, the position of the pelvis joint is tracked (being the parent joint of the skeleton data) to find its accuracy and RMSE with respect to the global frame. In addition, the RMSE of the position of the operator’s wrist is tracked to provide the error estimation with respect to the same global frame of reference. Finally, Kalman-based filtering is applied to fuse the data from the vision sensors at distinct collaborative zones assigned based on the analysed RMSE result. The authors also proposed a feasible control strategy of human motion tracking for Human Robot Collaboration (HRC) applications in a collaborative workspace.

## 2. State of the Art Review

This section presents recent publications that use human tracking systems based on vision sensors in collaborative environments. In most cases, the robot was fixed, and the human operator worked in proximity to complete independent tasks or interact with it. However, applications involving collaborative mobile robot and dual-arm mobile robots [25] exhibited a significantly increased complexity of the tracking strategy using vision systems due to occlusion, and various other hindrances.

A widespread application of such systems is ensuring that there is no collision between the end effector and a human or object and, to a lesser extent, between a human or object and the other joints of the robot. Bonci et al. [26] presented a proof of concept, dealing with the human-obstacle collision avoidance involving a collaborative fixed-base manipulator, utilising an Acusense RGB-D (Red Green Blue-Depth) camera. The collision avoidance strategy depended on the distance between the fixed robot and the operator. For short distances, it relied on the data collected from the depth sensor, while for longer distances (out of the range of the depth sensor) on the processing of the RGB frames using a You Only Look Once (YOLO)-based Convolutional Neural Network (CNN). Their proposed methodology claimed to reduce the amount of processed data while enhancing the operator’s safety. Scimmi et al. [27] approached the same problem using two Kinect v2 RGB-D cameras to acquire the position of the operator and avoid problems related to the occlusions of the sensors. Each camera extracted 25 joints of a human skeleton. The data collected from the two sets of coordinates were fused using a fusion algorithm developed to obtain the optimal skeleton poses. It was found that the proposed strategy could effectively alter the planned trajectory and prevent human–robot collisions in two case studies. Chen and Song [28] also used two Kinect V2 RGB-D cameras to develop a collision-free motion planning algorithm applied to a robotic arm. Initially, the acquired depth images were used to generate point cloud segmented objects, which were subsequently merged into a single cloud using a K-Nearest Neighbour (KNN) algorithm, aiming to identify the closest point from an obstacle to the robot. Moreover, a Kalman filter was applied in the process of estimating the obstacle motion parameters (velocity, position). It was found that the robotic manipulator managed to avoid collision with an obstacle and preserve the desired trajectory of the effector while following the proposed control design during a Cartesian hexagon task. Furthermore, Pupa et al. [29] applied an effective two-layered strategy for trajectory planning and velocity scaling in a six-DoF manipulator, aiming to enhance a safe HRC. The first layer planned dynamically the initial nominal trajectory, examined its feasibility at maximum velocity, and amended it based on human tracking information captured by six OptiTrack Prime cameras. The second layer adjusted the robot velocity to ensure that its limits adhered to ISO safety constraints. The system architecture was validated experimentally in two scenarios: when the operator hinders the motion or path of the robot and when the two agents are in proximity.

Several researchers have also investigated the use of cameras in conjunction with other sensors to implement dynamic obstacle avoidance strategies. For instance, Gatesichapakorn et al. [30] combined a laser localisation sensor with an RGB-D camera to navigate an autonomous mobile robot. The generation of the static map was implemented in the Robot Operating System (ROS) using a 2D laser-based Simultaneous Localisation and Mapping (SLAM) package. The experimentation in an indoor public space demonstrated the ability of the robot to adapt its motion to the appearance of a human obstacle and subsequently recover its trajectory. Another system that enabled the operation of an anthropomorphic robot through multiple sensors was proposed by Cherubini et al. [31] aiming to implement smart logistic tasks transporting automotive parts. It involved one RGB-D and four RGB cameras, two laser scanners, two force sensors, ten tactile sensors, and two stereo vision sensors where the individual tasks, including the target detection and obstacle mapping, were performed by different sensors. The robotic system was significantly accurate in recognising hand gestures, and therefore the authors proposed a real-time programming strategy based on sign language for intuitive robot control. It should be considered though that the use of such a high number of sensors increased the cost of the infrastructure significantly. Gradolewski et al. [32] presented a real-time safety system that proposes actions to a collaborative robot based on human detection and localisation. An HD vision camera was used for motion detection, together with an ultrasound sensor for proximity estimation. These devices, along with the controller, constituted the detection unit. Moreover, the authors estimated and compared three machine learning algorithms in terms of detection efficiency and maximum latency, concluding that YOLO outperformed Histogram of Oriented Gradients (HOGs) and Viola-Jones.

The improvement of the computational capabilities of Graphical Processing Unit (GPU) technology has significantly facilitated the integration of parallel computation into motion planning algorithms over the last years. Cefalo et al. [33] proposed an algorithm for collision detection to solve a Task-Constrained Motion planning problem [34], and applied it to a robotic arm. The proposed algorithm utilised two real-time images that presented the obstacle mapping (real depth image) and the future robot configuration (virtual depth image) obtained from a Kinect camera and the robot CAD model, respectively. The possibility of the collision scenario was processed in parallel by comparing the two images. Tölgyessy et al. [35] evaluated the Azure Kinect with its predecessors, namely, Kinect V1 and Kinect V2, focusing on precision and noise generation. Their study reported that the performance indicators of Azure Kinect lie within the range indicated in the official documentation. The study concluded that the Azure Kinect may not be suitable for outdoor applications due to limitations of the time-of-flight technology and requires a warm-up time of at least 40–50 min to give stable results.

Human pose detection with vision sensors is another key feature towards the enhancement of HRC activities. Johnson et al. utilised a vision-inertial-based fusion algorithm to initialise and calibrate a forward kinematic model of an arm, which tracks the position and orientation of the arm: the combination of using vision- and IMU-based sensors overcomes the drifts thereby improving the accuracy of tracking the pose of the human arm [36]. Similarly, a visual-inertial sensor-based approach with three sensor modules with each module comprising IMU and ArUco marker attached to three parts of the body mainly, to the trunk, upper arm, and forearm provides a simpler solution for the assessment of movement during robot-assisted training; the ArUco marker, which can be captured by the camera and the driftless orientation of the modules is computed via the visual-inertial sensor fusion algorithm [37]. An HRI framework using a vision-based system together with a three-axis accelerometer, trained on activity classification with a library of 22 gestures and six behaviours, demonstrated a 95% success in the recognition of gesture and 97% in the recognition of behaviour. The intelligent system integrates static and dynamic gestures using ANN and hidden Markov models [38]. Furthermore, a similar approach applied to a case study involving online robot teleoperation to assemble pins in car doors has been demonstrated [39]. An activity recognition strategy using Gaussian mixed HMM, using Microsoft Kinect, was able to detect the human activity with a recall accuracy of 84% with previously seen models and 78% with unseen models [40]. Also, Hernández et al. [41] compared the estimation of shoulder and elbow angles as captured by a webcam in rehabilitation exercises using markerless pose estimators from two CNN frameworks, OpenPose and Detectron2. The data collected from two Kinect V2 RGB-D cameras were fused to generate the ground truth for the upper body joint. OpenPose was found to identify the angles of the limbs more accurately than Detectron2 in all different scenarios. The tracking of the human body orientation with depth cameras, namely, Kinect V2, Azure Kinect, and ZED2i, for the detection of socially occupied space while interacting with people was investigated by Sosa-Leόn et al. [42]. Related approaches that identify the orientation of human body poses may be used in cases of Human–Robot Collaboration for real-time decision making and path planning to carry out tasks. Similarly, De Feudis et al. [43] assessed four different vision systems for hand tool pose estimation: ArUco, OpenPose, Azure Kinect Body Tracking, and YOLO network were used with HTC Vive as a benchmarking system. Further, in a study presented in [44], Azure Kinect and Intel RealSense D435i were compared where the Intel RealSense was reported to show poorer performance in the estimation after 2 m, while the Azure Kinect performed better. Furthermore, the study reported that the depth accuracy of Azure Kinect largely depends on the emissivity of the object, while the RealSense remained unaffected.

The experimentation involved three different motion scenarios of a human operator handling a cordless drill with its mandrel considered as the point of interest to be tracked [43]. The mean square point-to-point distance (D.RMS) and the multivariate R^2^ were used as the accuracy evaluation criteria. The authors found that the Azure Kinect Body Tracking attained the overall lowest performance, being particularly inaccurate to track the right- and left-hand joints. On the other hand, ArUco generated the most accurate results with the lowest standard deviation of D.RMS for all three scenarios. Similarly, another study [45] uses RGB data for task predictions within a collaborative workspace to manage an assembly process, which is validated by a demonstrator used to assemble a mechanical component. On evaluating four different frameworks, namely, Faster R-CNN, ResNet-50, and ResNet-101, YOLOv2 and YOLOv3, the YOLOv3 framework performed the best with an average mean performance of 72.26% when completing the assembly task.

## 3. Models and Methods

This paper proposes a new approach for comparing the performance of different vision systems, while taking advantage of the diverse capabilities of the associated hardware and software components, thus leading to the better human pose detection.

### 3.1. Experimental Setup

The skeleton pose detection was carried out using three depth-based vision sensors: Azure Kinect, Stereolabs ZED2, and Intel RealSense D455. Their key features are extensively presented in Table 1. The sensors were connected to a desktop computer with Intel i7-11th Gen 8 Core processor, 32 GB RAM, and 8 GB NVIDIA RTX 3070 graphic card. Each sensor uses a different depth-sensing technology. More specifically, AK utilises time of flight, i.e., emits and detects backscattered modulated light, translating the phase difference into depth distance for each pixel [46]. ZED2 uses a Convolutional Neural Network (CNN) algorithm for stereo matching [47], while Intel RealSense 455 [48] interprets the scene by comparing images acquired from two known and slightly different positions.

The markerless approach for skeleton tracking is primarily based on CNN approaches. Firstly, in the case of Azure Kinect, the Infrared Sensor (IR) data are fed into a Neural Network, which extracts a silhouette of users and 2D joint coordinates. Combining 2D joint pixel values with the depth data provides the 3D joint information of the skeleton joints [49]. Secondly, the ZED2 body tracking SDK uses neural networks to detect keypoints or the skeleton joints, which are combined with the depth and positional tracking provided by the SDK of ZED2 to obtain a 3D pose estimate of the persons in the scene. Finally, OpenPose, a popular pose estimation model [50] coupled with the Intel Realsense D455, is used to detect keypoints or parts to identify the human joints. Therefore, three sensors that are capable of skeleton-based tracking as well as of providing human key points [51,52,53] in 3D are used in this study.

A 2D pose estimation uses multi-stage CNN to predict Part Affinity Fields (PAFs) and confidence maps. The 2D joint pose estimation is converted into 3D information using depth data, if available [54]. The body tracking SDKs of Azure and ZED2 provide information about the individual joint positions and orientations, while in the case of the OpenPose framework [54] used in conjunction with the Intel D455, the skeleton information comprises exclusively 3D joint positions. Depending on the number of keypoints (joints) required, BODY_25 or COCO format could be chosen as the output of the OpenPose framework [55]. BODY_25 was preferred in this study as it attained faster detection by approximately 30% and higher accuracy by 3% compared to COCO [56]. In the case of the other two vision sensors, the default outputs of the SDK’s skeleton joint data were retained for the study. The authors individually compared the performances of the three body-tracking SDKs for the evaluation of the pose accuracy at different depths from the camera in order to find a suitable device that has the potential to be used in collaborative mobile robotic applications.

The options that the three depth cameras offer in terms of colour and depth resolution are presented in Table 2, along with the modes used in the current study. The experiments were performed within the ROS framework using the respective drivers of each sensor [57,58,59]. The joints information was acquired in the ROS network at a frequency of 18.5, 12, and 18 Hz for AK, ZED2, and Intel D455, respectively. In the case of AK, the NFOV (Narrow Field of View) mode with a range of 0.5–3.86 m was chosen for comparison with other vision sensors as NFOV covers more depth compared to WFOV (Wide Field of View) and attains superior pixel overlap as indicated by the manufacturer [60]. Furthermore, Tölgyessy et al. [9] tested various modes of AK body tracking SDK and reported that the data acquired using NFOV data were more stable than the WFOV mode. The resolution parameters selected for ZED2 and Intel D455 were based on the available computation power of the desktop computer and the requirement for the simultaneous operation of the three vision systems [61].

**Figure 1:**
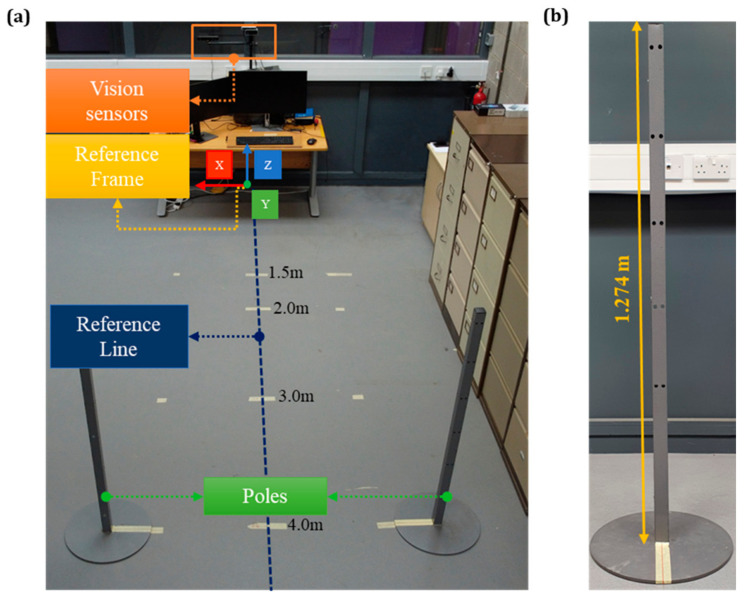
(**a**) Panoramic view of the laboratory with markings of discreet interval for estimating the pose of the operator at various depths, (**b**) the poles used for the pose estimation.

The experiments were carried out in a confined laboratory environment (7.8 × 3.4 × 4.5 m^3^) under physical lighting conditions involving natural sunlight and artificial roof light (Figure 1a). The various distance levels from the cameras (i.e., 1.5, 2.0, 3.0, 4.0, 5.0, 6.0 m) were marked on the reference line using a Bosch Laser Measure device (BLM) with ±1.5 mm (0.0015 m) accuracy to guide the operator. Moreover, two poles with a height of 1.274 m (Figure 1b) were placed on both sides of the reference line, serving as a guide for pose estimation involving the wrist joint.

**Figure 2:**
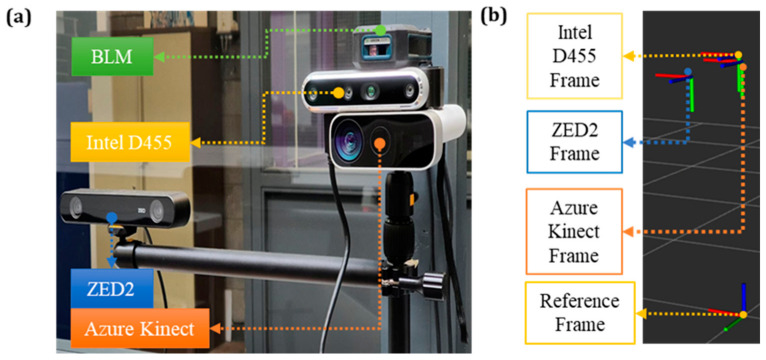
(**a**) Setup of the vision sensors on the desk camera mount, (**b**) the global reference frame and the frames of the vision sensors as depicted in RViz.

The three cameras and the BLM were clamped on a desk camera mount, as seen in (Figure 2a), ensuring that they were aligned to the XY plane. The data were acquired with respect to the global frame (Reference Frame), as shown in Figure 2b. According to the ROS conventions, the coordinate frames X, Y, and Z were represented in red, green, and blue, respectively. The global frame from RViz (visualisation tool in ROS) with the individual coordinates of Azure, Intel D455, and the ZED2 camera is shown in Figure 2b. The position of the coordinate frames of the cameras was measured using the BLM and was configured in the ROS launch files of each vision sensor to ensure the setup is similar in the real and the virtual world, i.e., by measuring the offset from the Reference Frame to AK, AK to Intel, and AK to ZED2.

**Figure 3:**
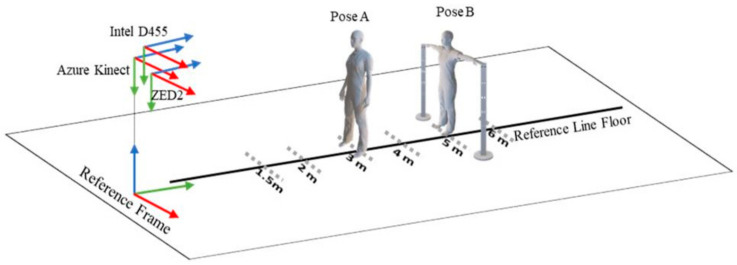
Experimental setup and procedure implemented to capture the poses of the operator at different depths.

After the initialisation of the cameras, the operator moved on each marked point, standing with the hands down (Figure 3, Pose A) and subsequently repeated the same with the wrist on top of the pole (Figure 3, Pose B). Next, the BLM device (Figure 2a) is connected to a smartphone via Bluetooth to estimate the distance between the camera and the operator (ground truth) and calculate the RMSE values.

Then, the cameras started to provide the skeleton joint coordinates published as ROS messages. Overall, 50 samples were collected for each camera, pose, and distance level.

The sequence of data collection was carried out as follows:

1. The vision sensor initialised, and the operator moved to the floor marker.
2. The operator recorded the ground truth depth using the BLM device.
3. The operator moved to Pose A, and the camera started to record the data. First, 50 samples of joint coordinates were collected (XYZ) from each device with respect to the global frame of reference.
4. The process was repeated for Pose B.

### 3.2. Skeleton Tracking Information

**Figure 4:**
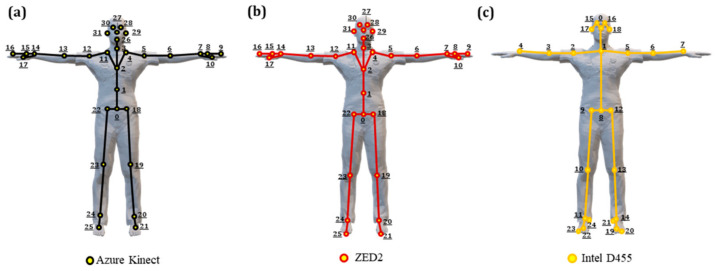
The skeleton joints with joint numbers shown in Table 3 below that can be tracked by (**a**) Azure Kinect, (**b**) ZED2, (**c**) Intel D455.

The skeleton joints available for tracking are shown in Figure 4, along with the corresponding names reported in Table 3 based on the documentation of the respective SDKs. Overall, AK, ZED2, and OpenPose provide skeleton data for 32, 34, and 25 joints, respectively. The joints that pertain to the eyes, ears, nose, the tip of the thumbs, and toes were not considered in the evaluation process as they do not affect or contribute to the operator’s pose (see Table 3).

**Figure 5:**
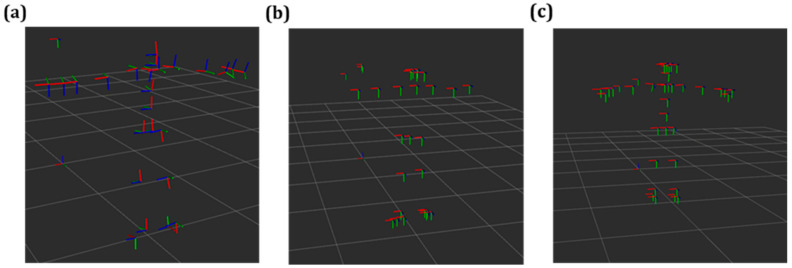
Transformation frames (TFs) of individual joints information in Rviz from vision sensor. (**a**) Azure Kinect, (**b**) Intel D455, (**c**) ZED2.

**Figure 6:**
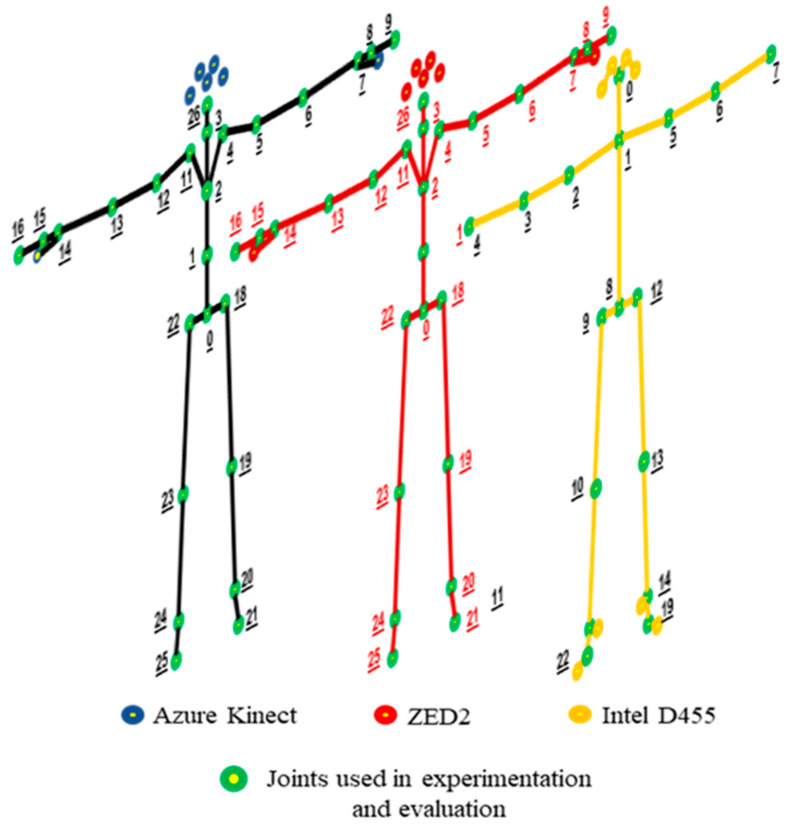
Illustration of the joints used for the evaluation of the three cameras.

Initially, the datatype acquired from the SDKs via the ROS drivers of AK, ZED2, and Intel D455 was analysed. It was noted that the data of joints belonged to two different types, i.e., MarkerArray in the case of AK and List in the case of ZED2 and Intel D455. Therefore, it was processed and published as TF frames, as shown in Figure 5, for the calculation of translation (X, Y, Z) and rotation (quaternion or roll, pitch, and yaw) of various joints with respect to the reference frame (Figure 2b). Each of the joints used for evaluation in this study is shown in Figure 6.

At a distance of 1.5 m, the Intel D455 camera could capture only the upper body joints (pelvis included) (Table 1) due to the restricted field of view of the captured image data. However, in the case of AK and ZED2, the body tracking algorithm could predict the position of the lower joints of the operator and provide information with low accuracy. Furthermore, as the operator moved further away from the cameras (>1.5 m), the joints below the pelvis were also visible.

Apart from tracking the overall skeleton, particular focus was given to the tracking accuracy of the pelvis and wrist (right and left) joints. The reason is that the pelvis is the first parent joint of the skeleton pose; therefore, its accuracy and stability are critical. In addition, the tracking stability of the wrist joints is important, especially in the case of extension of the limbs (e.g., Pose B), and should be primarily considered when the HRC’s effectiveness is assessed.

### 3.3. Preliminary Test—Evaluation of Raw Data

**Figure 7:**
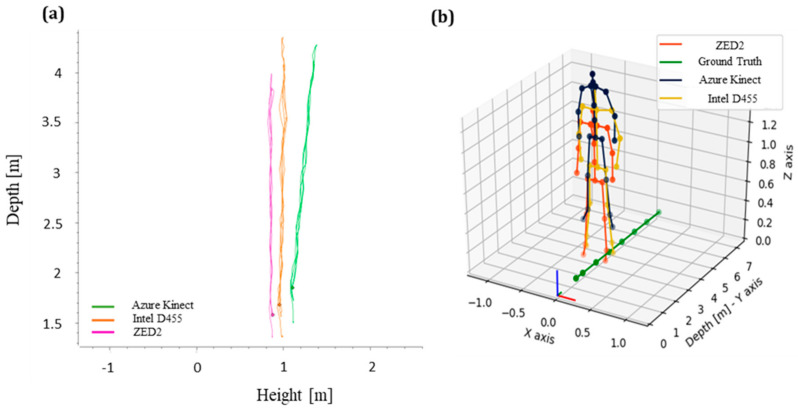
(**a**) Relation between the height (Z) and depth (Y) of pelvis joint, (**b**) corresponding skeleton joint data from each camera at a distance level of 3 m (plot view−upper elevated angle).

After the camera setup, a preliminary procedure was devised to test the raw data. It was observed that during the tracking of joints in RViz, as the operator moved away from the camera, the skeleton gradually levitated from the ground in the case of AK (see Figure 7).

To investigate this further, additional tests were performed with the pelvis joint being tracked while the operator was moving along the reference line, starting from a distance of 1.5 m. In this way, the height (Z)–depth (Y) plot was defined (Figure 7a) with a noticeable slope to be observed exclusively in the case of AK. The skeleton poses are shown indicatively in Figure 7b for a distance level of 3 m. It can be observed that the skeleton coordinates acquired by AK are higher than the respective ZED2 and Intel D455.

The corresponding slope was analytically estimated at −0.110417. As a result, the final height (Z′) obtained by the AK coordinated data was calculated based on the real-time values of Z and Y as updated within the published TF data using Equation (1):

(1) $$Z^{\prime }=Z-slope\times Y$$

**Figure 8:**
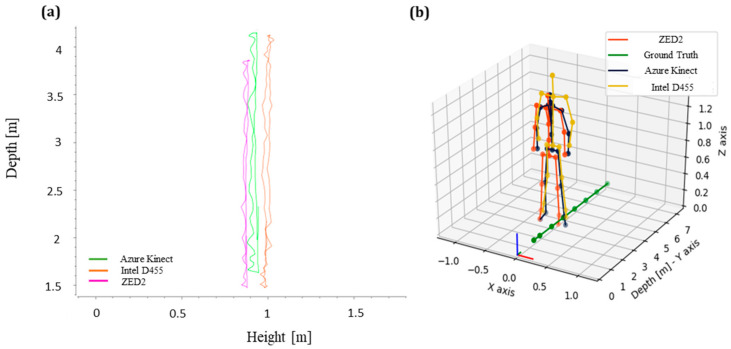
(**a**) Relation between the height (Z) and depth (Y) of the pelvis joint after the slope compensation, (**b**) corresponding skeleton joint data from each camera at a distance level of 3 m.

In addition, a moving average filter with a window size of 30 data was applied to the real-time data to minimise the noise. The obtained results are shown in Figure 8a, where the slope of Azure Kinect is significantly reduced, while the respective skeleton poses are shown in Figure 8b, with the pelvis joint of AK closely aligned with the pelvis joints of the other vision sensors.

## 4. Results and Discussion

This section assesses the accuracy of the depth (Y) estimation for the three cameras resulting from the tracked skeleton joint and evaluates their performance while capturing the two poses at various depths (distance levels from the camera). Two data sets are presented: (i) the raw skeleton data from the cameras in Section 4.1 and (ii) the filtered data (after applying the moving average filter) in Section 4.2. In both cases, the AK slope was compensated to minimise the levitation from the ground, as previously explained (Section 3.3). For the further evaluation of Pose B, the left and right wrists were selected as the common joints of all three cameras (see Table 3, joint numbers: Azure Kinect: 7, 14, ZED2: 7, 14, and Intel D455: 4, 7).

### 4.1. Accuracy Estimation of the Raw Data

#### 4.1.1. Evaluation of the Depth Accuracy

**Figure 9:**
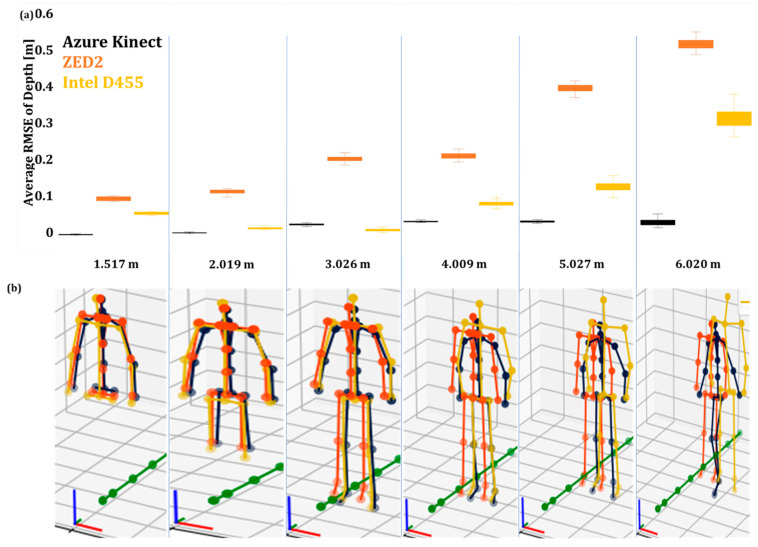
The unfiltered joint data of the three vision sensors capturing the human skeleton in Pose A: (**a**) average RMSE values of unfiltered joint data with a 3D skeleton at different depths, (**b**) the tracked skeleton joints of the operator at the various depth values.

**Figure 10:**
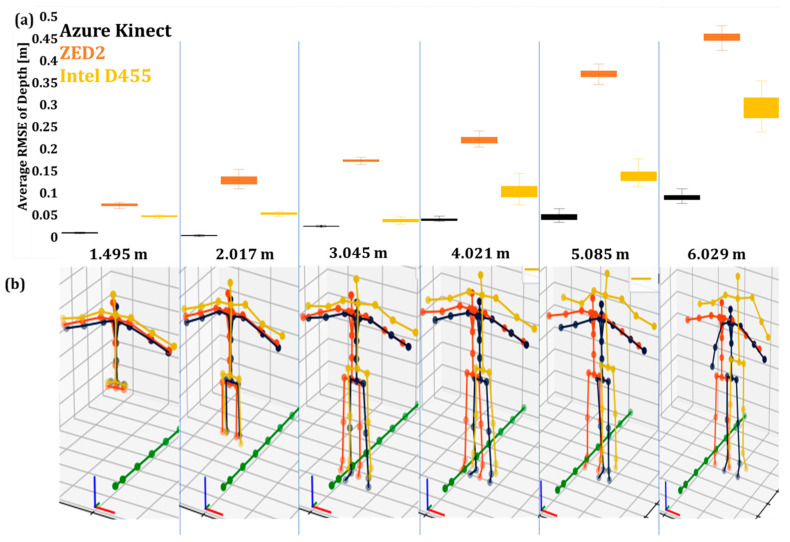
The unfiltered joint data of the three vision sensors capturing the human skeleton in Pose B: (**a**) average RMSE values of unfiltered joint data with a 3D skeleton at different depths, (**b**) the tracked skeleton joints of the operator at the various depth values.

The average RMSE values of the operator’s depth (Y) in Pose A for 50 iterations are shown in Figure 9a, while Figure 10a presents the results for the operator in Pose B.

The box plots showed that the AK body tracking attained the lowest RMSE, followed by Intel D455 and ZED2 (Figure 9a and Figure 10a). This increase in the average RMSE may be due to the inverse relationship between the disparity-depth pixel information [62]. Furthermore, the perspective foreshortening effect may have affected the accuracy of the skeleton poses in the case of stereo cameras [63,64,65]. Moreover, as the operator moved further away from the camera, the AK and Intel D455 joint data became unstable, and the deviation of the acquired skeletons from the original poses became significant at distances higher than 4 m (Figure 9b and Figure 10b). On the other hand, in the case of ZED2, the acquired skeleton was relatively consistent for both poses and all distance levels.

#### 4.1.2. Overall Performance of the Skeleton Pose Estimation—Pose A and Pose B

**Figure 11:**
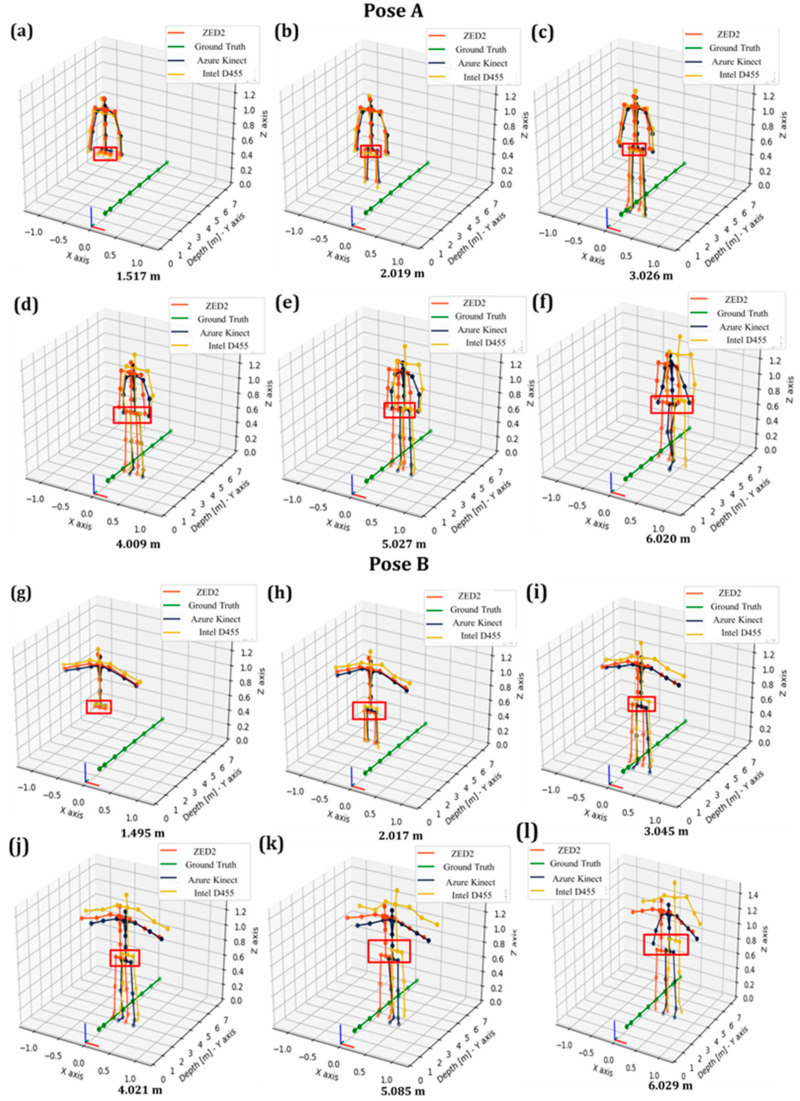
Evaluation of RMSE of pelvis joint data in Poses A and B with deviation of pelvis joint along the depth axis from the vision sensors in the range from 1.5 m to 6.0 m.

The tracking of the overall skeleton joints obtained from the three sensors is depicted in Figure 11.

In Figure 11, as obtained from all vision sensors, it was noted that the overall skeleton poses of the operator presented gradual deviation along the *X*-axis with respect to the global frame, as indicated by the red rectangle on the pelvis joint. This trend was obtained for Pose A (Figure 11a–f) and Pose B (Figure 11g–l).

**Figure 12:**
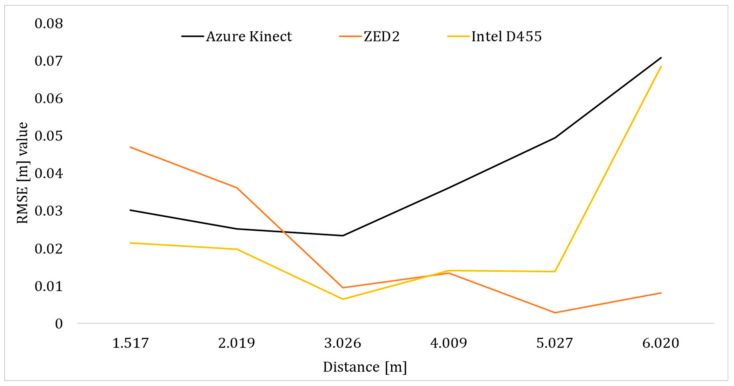
RMSE of the unfiltered pelvis joint position along the *X*-axis for the three vision sensors in Pose A.

In the case of ZED2 and for both poses, the X-RMSE reduced as the operator moved away from the camera, as depicted in Figure 12 indicatively for Pose A. More specifically, the RMSE of the unfiltered pelvis joint data from AK and Intel D455 was lower than ZED2 by approximately 43% and 74%, respectively, at depth ranges of less than 2.5 m. However, at higher depths (>3 m), ZED2 demonstrated superior tracking performance in this distance range especially considering the tendency of AK and Intel D455 to deform the tracked skeleton significantly (Figure 9 and Figure 10).

#### 4.1.3. Pose Accuracy Estimation by Tracking Wrist Joint—Pose B

**Figure 13:**
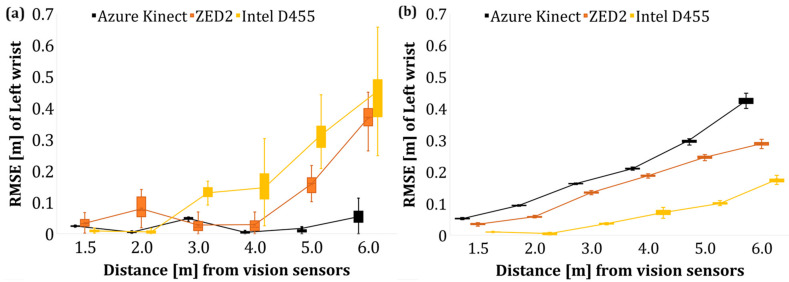
RMSE of left wrist joint of the operator in Pose B. (**a**) *Y*-axis (depth) data, (**b**) *Z*-axis (height) data.

**Figure 14:**
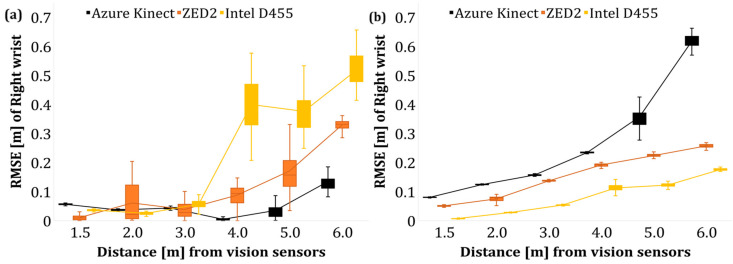
RMSE of right wrist joint of the operator in Pose B. (**a**) *Y*-axis data, (**b**) *Z*-axis data.

Following the evaluation of the overall skeleton of the operator in Poses A and B, an additional evaluation was performed to estimate the position of the wrist joint using the poles as fixed objects, i.e., with known positions with respect to the reference line. As a result, the RMSE of the Y and Z for the discrete distance levels is presented in Figure 13 and Figure 14 for the left and right wrist, respectively.

Overall, an increase in the average RMSE of the wrist joint was observed as the operator–sensor distance increased. The deterioration of the tracking accuracy of the limbs with the tracking distance has also been confirmed by Romeo et al. [66] who reported that the acquired data of AK that pertained to the limbs (wrist, hands) were less accurate compared to the data of the upper body joints such as the pelvis, chest, and neck.

As the vision sensors utilise similar AI-based body tracking approaches to train their data, the results of ZED2 and Intel D455 resemble AK data. Training AI-based pose estimation neural networks with synthetic data in realistic conditions accounting for various extrinsic factors, image disparity, occlusion, and foreshortening may improve the overall accuracy of pose estimation.

### 4.2. Accuracy Estimation of the Filtered Data

This section shows the results of the second data set, filtered in real time using a moving average filter to minimise noise, jitter, and outliers.

#### 4.2.1. Evaluation of the Depth Accuracy

**Figure 15:**
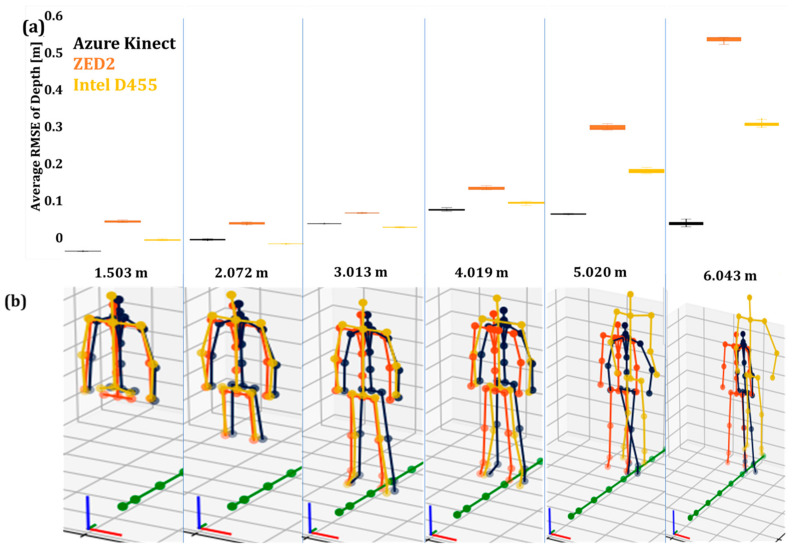
The filtered joint data of the three vision sensors capturing the human skeleton in Pose A: (**a**) average RMSE values of unfiltered joint data with 3D skeleton at different depths, (**b**) the tracked skeleton joints of the operator at the various depth values.

**Figure 16:**
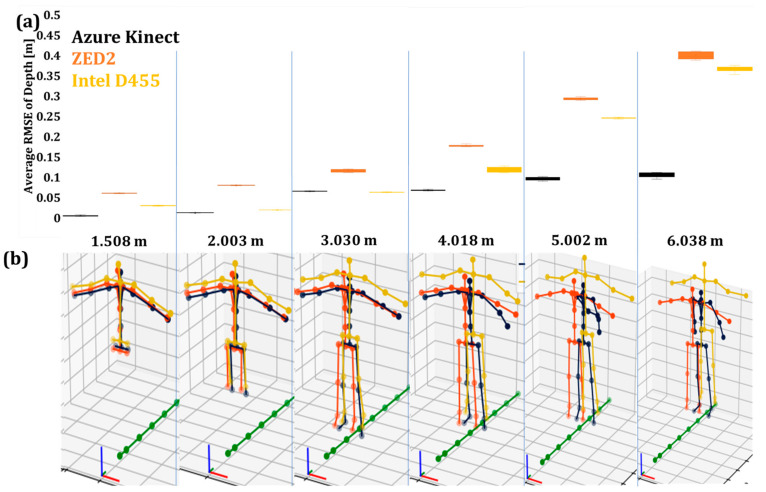
The filtered joint data of the three vision sensors capturing the human skeleton in Pose B: (**a**) average RMSE values of unfiltered joint data with 3D skeleton at different depths, (**b**) the tracked skeleton joints of the operator at the various depth values.

The average RMSE values of the operator’s depth (Y) in Poses A and B after data filtering are presented in Figure 15a and Figure 16a. Figure 15b and Figure 16b present the overall posture of the skeleton in the two poses. In general, RMSE follows the same trend with the unfiltered data, i.e., increases as the camera–operator distance increases. Moreover, the filter had an overall positive effect on the capturing of Pose B in the case of Intel D455 and a negative in the case of AK, especially at longer distances. ZED2 had consistent skeleton tracking in most cases.

#### 4.2.2. Overall Performance of the Skeleton Pose Estimation—Pose A and Pose B

**Figure 17:**
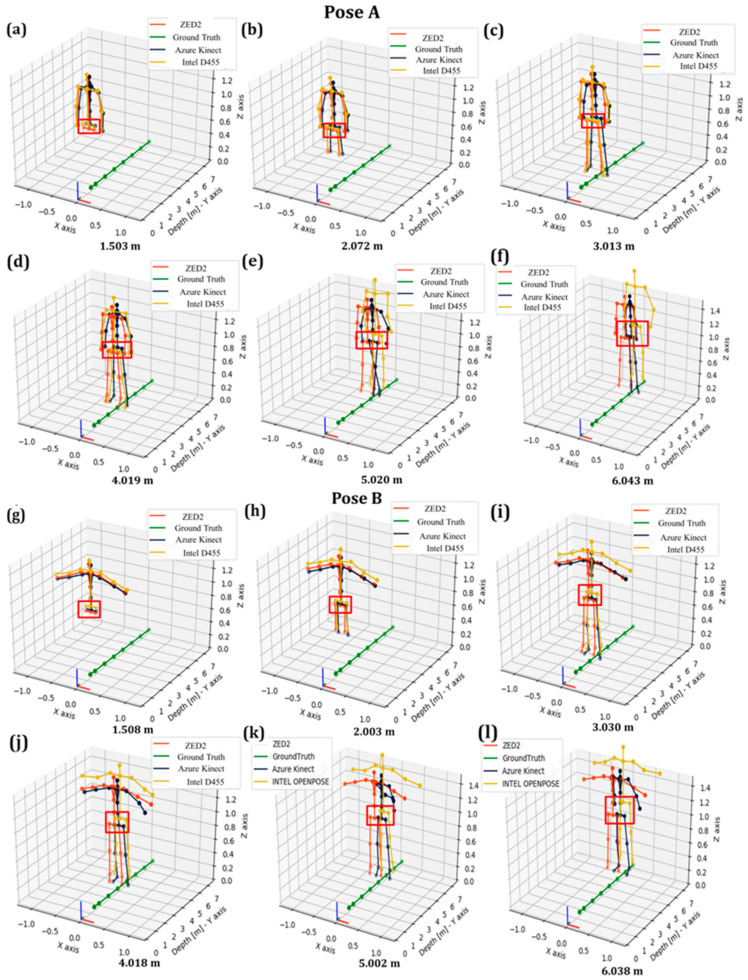
Evaluation of RMSE of pelvis joint in Poses A and B with deviation of pelvis data along the depth axis from vision sensors in the range from 1.5 m to 6.0 m.

The 3D plots of the overall skeleton poses are presented in Figure 17.

It can be deduced that the operator’s pose shifts gradually toward the positive X as it moves further away from the camera, similar to the results of unfiltered pelvis joint data as presented in Figure 11. Nevertheless, in the case of filtered data, the skeleton shift appears to take place more gradually. This effect may occur due to the minor standard deviation in the filtered data explained in the following section, which compares the raw and filtered data results.

**Figure 18:**
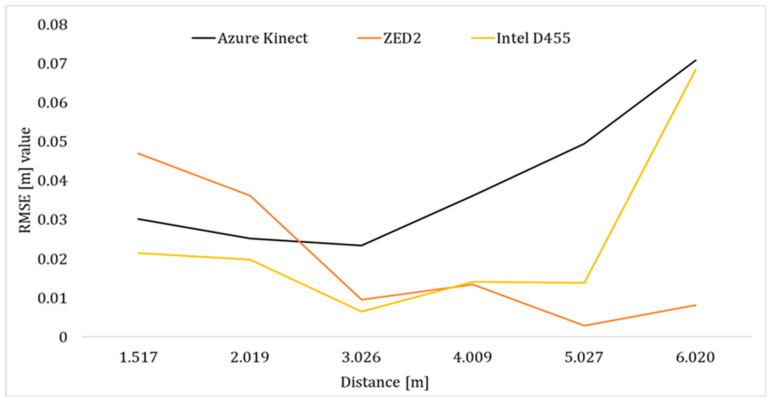
RMSE of the filtered pelvis joint position along the *X*-axis for the three vision sensors in Pose A.

The X-RMSE curve of the pelvis joints (Pose A) for Intel D455 was higher than AK and ZED2 (see Figure 18). Therefore, it can be stated that applying a real-time filter to Intel D455 data did not contribute to the reduction in its X-RSME values, while it lowered the error in the case of AK (see also Figure 12). Also, beyond 4 m, the tracking of the pelvis joint became unstable in the case of AK and Intel D455. In the case of AK, this may happen due to the limitations of the hardware’s tracking capabilities. The significant increase in the filtered X-RMSE of Intel D455 may have been caused due to an external disturbance that pertains to the extrinsic conditions of the laboratory, leading to poor accuracy. For instance, certain settings of the Intel D455 camera were not modified, e.g., the exposure was set to auto mode. However, this does not impact the tracked depth but affects the quality of the output image [67]. Furthermore, since OpenPose is primarily a 2D pose estimation algorithm, which uses colour images, this may have impacted the X-RMSE value.

#### 4.2.3. Pose Accuracy Estimation by Tracking Wrist Joint—Pose B

**Figure 19:**
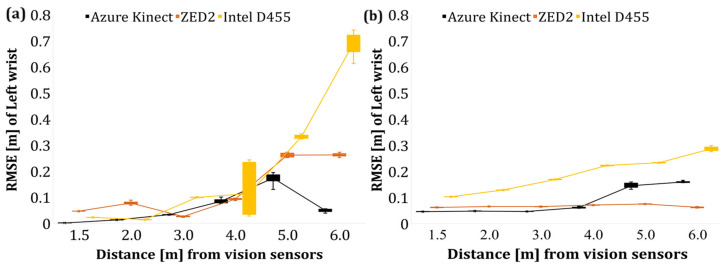
RMSE of left wrist joint of the operator in Pose B (filtered). (**a**) *Y*-axis (depth) data, (**b**) *Z*-axis (height) data.

**Figure 20:**
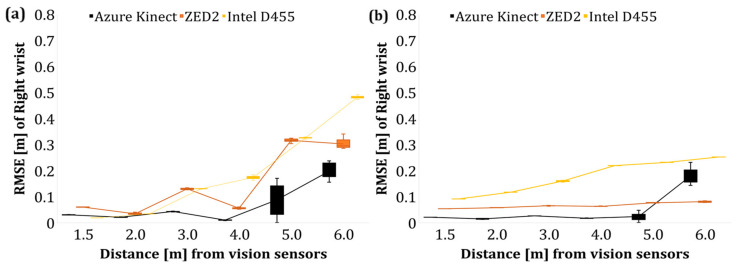
RMSE of right wrist joint of the operator in Pose B (filtered). (**a**) *Y*-axis (depth) data, (**b**) *Z*-axis (height) data.

The RMSE of the operator’s left and right wrist joints after the data filtering is shown in Figure 19 and Figure 20, respectively. The application of a low pass filter, such as a moving average filter, reduced the error of the wrist joints with respect to the *Y*- and *Z*-axis by lowering the random noises that affect the acquired data in the case of AK and ZED2. However, in the case of Intel D455, the overall RMSE is much higher than AK and ZED2, which indicates a minor effect of the applied filter, which extrinsic factors may cause during the tests. In addition, the postprocessing filter, namely, the temporal filter, was applied to the RealSense data configured in the camera’s ROS initialisation file. Therefore, the extrinsic factors and the postprocessing filter may have had no effect on reducing the overall RMSE value in the case of Intel D455. However, fine tuning the postprocessing filters under controlled light settings may reduce the RMSE error of the Intel D455 camera.

### 4.3. Unfiltered vs. Filtered Data

**Figure 21:**
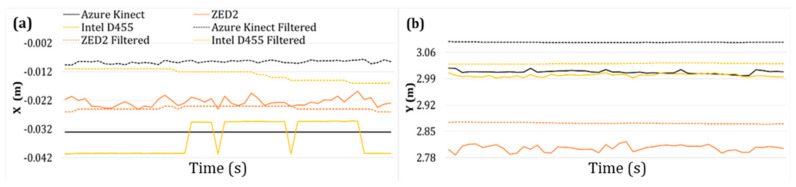
Differences in filtered and unfiltered data of pelvis joint at 3 m: (**a**) *X*-axis data, (**b**) *Y*-axis data.

This section presents a comparison of the raw and filtered data. For example, the X and Y values of the operator’s pelvis joint at a depth of 3 m are presented in Figure 21, before and after applying a filter. The significance of its application is indicated by the conversion of the raw (noisy) data curve to a smooth (filtered) curve in the case of all vision sensors.

**Figure 22:**
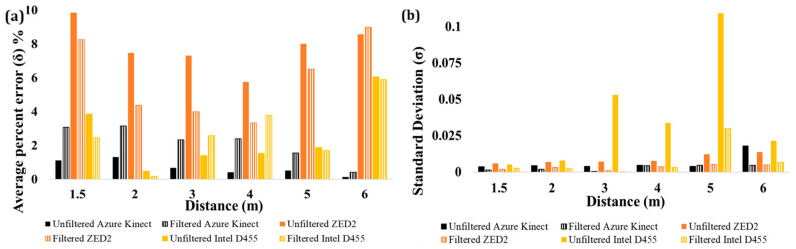
(**a**) Absolute percent error (δ) of average depth measurement of joints in Pose A, (**b**) standard deviation (σ) of the average depth data of skeleton joints in Pose A.

The authors also estimated the percent error (δ) of the depth (Y) at all distance levels in Pose A. The obtained results are reported in Figure 22. For Intel D455 and ZED2, δ was estimated at less than 2% at 4 m (Figure 22a), indicating its compliance with the respective values reported by the product specification [68,69]. In the case of ZED2, at short distances, the estimated δ was slightly higher than the one reported by the manufacturer.

Although data filtering has slightly increased the RMSE in the estimation of overall poses, its application in HRC scenarios may be preferable due to the resulting reduction in the Standard Deviation (σ) (Figure 22b).

**Figure 23:**
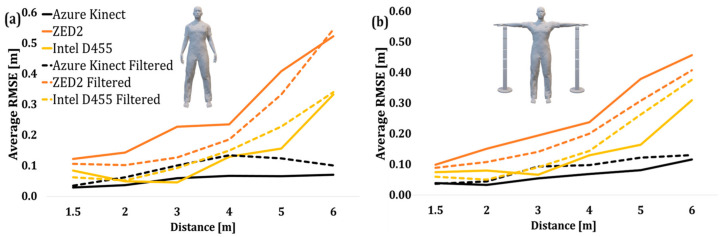
Average RMSE of joint depth values of two poses before and after applying moving average filter. (**a**) Pose A, (**b**) Pose B.

The average RMSE values of the overall joint depth data of the operator in Poses A and B are depicted in Figure 23.

**Figure 24:**
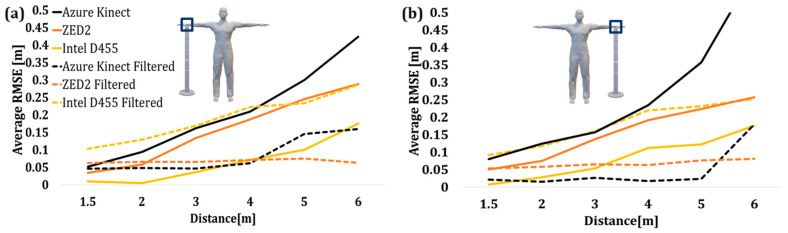
Average RMSE of joint height values of the wrist data before and after applying moving average filter. (**a**) Left wrist, (**b**) right wrist.

Similarly, the average Z-RMSE values of the wrist joints before and after filtering are shown in Figure 24. In this case, as the operator–camera distance increased, there was an increase in the overall average RSME data. The applied filter significantly improved the AK data compared to the rest of the output of the sensor, followed by ZED2.

### 4.4. Data Fusion in the Collaborative Zones

Based on the results obtained from the assessment of the performance of the cameras, the authors defined three collaborative zones and proposed a Kalman-based sensor fusion approach to combine the joint data and reconstruct the skeleton pose of the operator. The proposed approach was tested with the operator in Pose A.

#### 4.4.1. Classification of Collaborative Zones and Sensor Fusion

**Figure 25:**
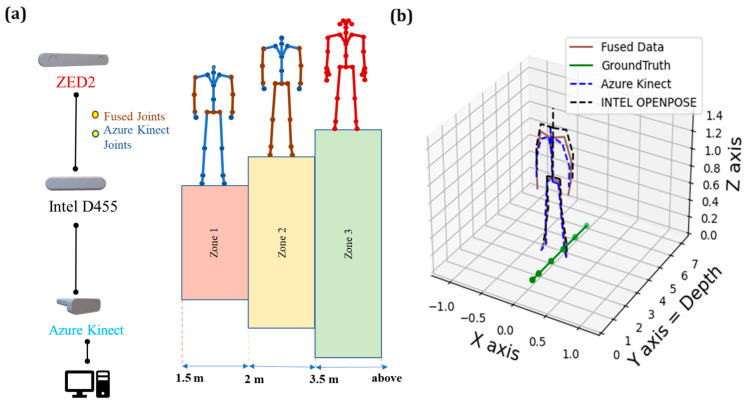
(**a**) Classification of zones for HRC tasks using multiple vision-based tracking systems, (**b**) example of fused output—Zone 1 at 1.8 m.

The design of collaborative zones aimed to minimise the error of the joints and facilitate a safer HRC. Therefore, they were classified as Zone I (1.5 m to 2.0 m), Zone 2 (2.0 to 3.5), and Zone 3 (3.5 m and beyond), depending on the distance from the vision sensors, as presented in Figure 25. These limitations were defined considering the capabilities of the vision sensors as reported by the manufacturers (see also Table 1).

In Zones 1 and 2 (Figure 25a), 23 joints were available to reconstruct the skeleton pose using the data obtained from both AK and Intel D455, as they demonstrated a better performance in depths of this range (see Figure 9 and Figure 15). The common joints (indicated in brown) were fused using a Kalman filter, while the rest (shown in blue in Figure 25) were used as obtained from the AK. In Zone 3, ZED2 was explicitly used to track the skeleton pose (shown in red in Figure 25) due to its capability to track accurately at far distances, as explained in Section 4.1 and Section 4.2.

#### 4.4.2. Pose Accuracy Estimation of Fused Data in the Collaborative Zone

**Figure 26:**
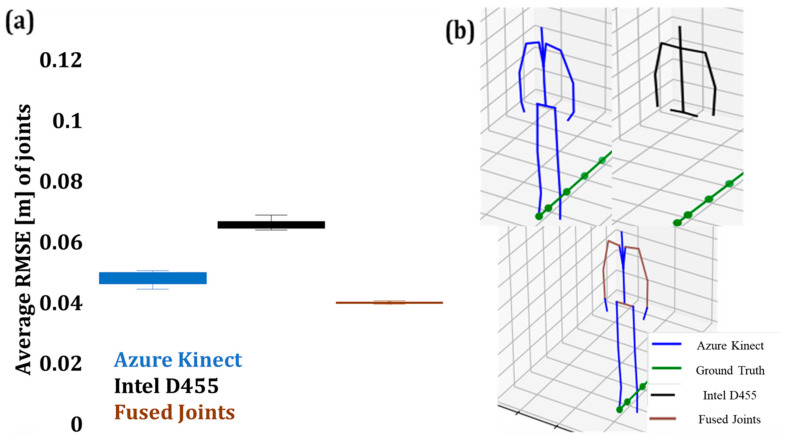
(**a**) RMSE of fused joint depth values in Zone 1 with the corresponding skeleton pose from AK (blue), Intel (black), and combined skeleton (brown), (**b**) fused and reconstructed skeleton joints.

This section provides the box plot of the results after the Kalman-based fusion of joints in the collaborative workspace. Figure 26a illustrates the average RMSE of the joint data derived from AK and Intel D455 and the RMSE of nine fused joints in Zone 1, which appears to be the lowest. The fused average RMSE of the joint depth values was estimated at 0.0389 m, with AK and Intel D455 values at 0.0472 m and 0.0649 m, respectively. Figure 26b shows the skeleton pose of the operator at approximately 1.826 m from the camera with AK, Intel D455, and fused joints to be depicted in blue, black, and brown, respectively.

**Figure 27:**
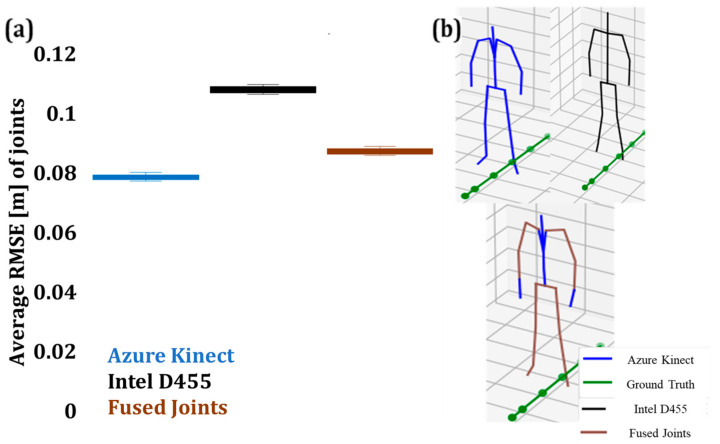
**(a)** RMSE of fused joint depth values in Zone 2 with the corresponding skeleton pose from AK (blue), Intel (black), and combined skeleton (brown), (**b**) fused and reconstructed skeleton joints.

Similarly, Figure 27 illustrates the RMSE of the skeleton joints in Zone 2 with 15 joint data fused in the case of Pose A. The common joints were analytically listed in Table 3.

At a distance of 2.699 m (Zone 2), the average RMSE values from AK and Intel D455 were 0.0784 m and 0.1078 m, respectively, while the RMSE corresponding to the fused joints was 0.08721 m (see Figure 27). This increase in the error of the fused joints may be caused due to extrinsic conditions. However, with further tuning of the sensors’ parameters, such as exposure, resolution, and noise filtering, as well as with the application of available postprocessing techniques, this error may be further reduced.

## 5. Conclusions

This study aimed to determine the accuracy of skeleton pose estimation at various depths using three different commercial vision systems and frameworks of skeleton pose tracking. One of the goals of the study was to compare various spatial computation kits, which differ in terms of hardware devices and associated software frameworks for tracking human operators. The comparison focused on evaluating the devices and frameworks leading in terms of human operator pose accuracy.

Based on the obtained results, the performance of the sensors from highest to lowest (in the order depth tracking: closer to far distance range) was assessed as follows: AK, Intel D455, and ZED2. The initial evaluation of the raw pelvis data demonstrated that AK data showed a linear levitation trend in height (Z) of the skeleton pose as the operator–camera distance increased. An analytical approach was used to minimise this slope. The obtained results showed that as the operator–camera distance increases, the skeleton pose gradually transitions with respect to the global frame. This phenomenon may affect safety and may be crucial in HRC applications. The deployment of multi-vision-based tracking systems can contribute to the minimisation of such an error.

Comparing the depth accuracy of raw and filtered data, it can be inferred that at a range shorter than 3 m, the AK and Intel D455 demonstrated better performance than ZED2, with the latter providing better tracking results beyond this range. However, at a distance approximately higher than 3.5 m, the tracking of AK becomes unstable due to the constraints of the NFOV mode, which has an operating range of 3.86 m. Therefore, when it comes to detecting entire skeleton poses beyond the range of 3.5 m, it is safer to utilise ZED2 to track entire human body poses and use bounding boxes. Further, with the additional functional tracking features, such as the velocity of the human operators, provided by the SDK of ZED2, this information can be easily used for collaborative mobile robotic applications for long-range tracking in shopfloor environments.

The tracking accuracy relies on various extrinsic parameters, such as the lighting conditions, the colour of the dress or jackets worn by the operator, the background colour, the resolution of the cameras, and the available computational power. Also, more variable parameters of the vision sensor are involved when multiple sensors are present in a scene. In addition, installing different SDKs and dependencies packages can be tedious and may lead to longer building time and runtime errors. Hence, extra attention should be given while performing these tasks involving different configurations of CMake flags, CUDA, and cuDNN versions. Furthermore, as more operators may be present in the scene, the computer requires more processing power to detect the operators’ skeleton joints without compromising the FPS rate.

Finally, developing such sophisticated algorithms utilises different software libraries (open source or commercially licensed), software packages, tools, etc., that contain thousands of lines of codes that have been independently tested. Hence, in the cases of deployment of various spatial computation frameworks, a constant tracking of updates is required in order to keep up to date with the latest features and functionalities provided by the SDKs. For instance, Sterolabs (ZED2) provided more frequent software updates with features and bug fixes, which in turn enhances the performance of the vision sensors.

When deployed, the capabilities of AI-based tracking of the human operator, on the whole, may vary in each scenario; this can be a risk and one of the significant challenges to consider when deploying similar tracking solutions, especially when compared against more conventional, safety-certified solutions.

As AI markerless tracking demonstrates moderate results regarding accuracy, its use on the workspace of a shop floor and its adoption by manufacturing companies is still limited. Their deployment in HRC scenarios in conjunction with additional ISO-certified safety sensors is still preferred in industry. Along with the skeleton tracking, additional features of SDKs, such as the object detection module of ZED2 SDK, may be used to determine the bounding box, the absolute velocity, and the operator’s position. The data collected could be used in conjunction with a sensor data processing or fusion algorithm, such as the Kalman filter or the particle filter algorithm to localise the operator’s position within a collaborative workplace. In addition, the position and velocity information could be used to sync the movements of a collaborative mobile robot with the movement of a human operator. As the pelvis is the parent joint that connects the rest of the skeleton joints, additional work in this area could involve its marker-based tracking in order to improve the overall skeleton accuracy. Future work includes the setup of a controlled lighting condition with LED and testing the performance of the vision sensor under various settings such as resolution, FPS, and brightness. Other classical or machine learning-based methods of determining the position of the human body, including, for instance the use of the pictorial structure framework approach or deep learning methods, could also be tested and benchmarked in the future using diverse hardware or software configurations.

## Figures and Tables

**Table 1:** Comparison of the depth sensor specifications.

	Azure Kinect	ZED2	RealSense D455
Released date	June 2019	October 2020	October 2020
Price	EUR 370	EUR 463	EUR 432
Depth sensing technology	Time of flight	Neural Stereo Depth Sensing	Stereoscopic
Body tracking SDK	Azure Kinect Body Tracking SDK	ZED Body tracking SDK	OpenPose v1.7.0 Framework
Field of view (depth image)	NFOV unbinned 75° × 65°	110° × 70°	87° × 58°
Specified measuring distance	NFOV unbinned 0.5–3.86 m	0.3–20 m	0.6–6 m

**Table 2:** The colour and depth resolution of the cameras used in the experiments.

	Azure Kinect	ZED2	Intel RealSense D455
SDK Version	1.1.0	3.7.1	v2.50.0
Colour resolution	640 × 576 @ 30 fps	720p @ 30 fps	640 × 480 @ 30 fps
Depth resolution/mode	NFOV unbinned 640 × 576 @ 30 fps	Ultra	640 × 480 @ 30 fps

**Table 3:** The skeleton joints are tracked from various cameras.

Joint No.	Azure Kinect	ZED2	Intel RealSense D455
0	Pelvis ′,″	Pelvis	Nose *
1	Spine Naval	Naval Spine	Neck
2	Spine Chest	Chest Spine	Right Shoulder ′,″
3	Neck	Neck	Right Elbow ′,″
4	Clavicle Left	Left Clavicle	Right Wrist ′,″
5	Shoulder Left ′,″	Left Shoulder	Left Shoulder ′,″
6	Elbow Left ′,″	Left Elbow	Left Elbow ′,″
7	Wrist Left ′,″	Left wrist	Left Wrist ′,″
8	Hand Left	Left Hand	Mid Hip (Pelvis) ′,″
9	Handtip Left	Left Handtip	Right Hip ′,″
10	Thumb Left *	Left Thumb *	Right Knee ″
11	Clavicle Right	Right Clavicle	Right Ankle ″
12	Shoulder Right ′,″	Right Shoulder	Left Hip ′,″
13	Elbow Right ′,″	Right Elbow	Left Knee ″
14	Wrist Right ′,″	Right Wrist	Left Ankle ″
15	Hand Right	Right Hand	Right Eye
16	Handtip Right	Right Handtip	Left Eye
17	Thumb Right *	Right Thumb *	Right Ear
18	Hip Left ′,″	Left Hip	Left Ear
19	Knee Left ″	Left Knee	Left Big Toe
20	Ankle Left ″	Left Ankle	Left Small Toe *
21	Foot Left ″	Left Foot	Left Heel ″
22	Hip Right ′,″	Right Hip	Right Big Toe
23	Knee Right ″	Right Knee	Right Small Toe *
24	Ankle Right ″	Right Ankle	Right Heel ″
25	Foot Right ″	Right Foot	Background *
26	Head	Head	-
27	Nose *	Nose *	-
28	Eye Left *	Left Eye *	-
29	Ear Left *	Left Ear *	-
30	Eye Right *	Right Eye *	-
31	Ear Right *	Right Ear *	-
32	-	Left Heel *	-
33	-	Right Heel *	-

′ Common joints in Zone 1 fused using Kalman filter; ″ common joints in Zone 2 fused using Kalman filter; * joints excluded from the overall experiment as they do not affect the skeleton pose.

## Data Availability

Data are contained within the article.
